# Optical Hydrogel Detector for pH Measurements

**DOI:** 10.3390/bios12010040

**Published:** 2022-01-13

**Authors:** Yousef Alqurashi, Mohamed Elsherif, Asail Hendi, Khamis Essa, Haider Butt

**Affiliations:** 1 Department of Mechanical Engineering, School of Engineering, Shaqra University, Dawadmi 11921, Saudi Arabia; 2 Department of Mechanical Engineering, Khalifa University, Abu Dhabi 127788, United Arab Emirates; mohamed.elsherif@ku.ac.ae; 3 School of Engineering, University of Birmingham, Birmingham B15 2TT, UK; asailhendi@outlook.com (A.H.); k.e.a.essa@bham.ac.uk (K.E.)

**Keywords:** sensors, pH-responsive hydrogels, Fresnel lens, hydroxyethyl methacrylate

## Abstract

Measuring pH has become a major key for determining health conditions, and food safety. The traditional pH assessment approaches are costly and offer low sensitivity. Here, a novel pH sensor based on a pH-responsive hydrogel has been developed. A Fresnel lens pattern was replicated on the surface of the pH-responsive hydrogel using the replica mould method. The pH sensors were tested in a pH range of 4–7. Introducing various pH solutions to the pH sensor led to volumetric shifts as the hydrogel swelled with pH. Consequently, the dimensions of the replicated Fresnel lens changed, modifying the focal length and the focus efficiency of the optical sensor. As a result, the measured optical power at a fixed distance from the sensor changed with pH. The optical sensor showed the best performance in the acidic region when pH changed from 4.5 to 5.5, in which the recorded power increased by 13%. The sensor exhibited high sensitivity to pH changes with a short respond time in a reversible manner. The developed pH optical sensor may have applications in medical point-of-care diagnostics and wearable continuous pH detection devices.

## 1. Introduction

Heart disease is a chronic condition that continues to be the primary cause of death in Europe [1]. Improved treatment methods for such disorders have been under review. Monitoring the changes in pH of blood is one way to prevent many fatal implications [2,3]. pH has become a major signal for determining health conditions, and food safety. In food-quality applications, variations in pH are generally correlated with bacteria growth and colonisation. In human blood, an increase in the pH level beyond the normal range (7.35–7.45) is related to degenerative diseases, and a decrease in the pH level is related to lower blood flow due to excessive acid adhering to the vessel [4,5,6]. The pH determination is necessary in a variety of applications in many fields such as chemistry and medical research [7].

Hydrogels are three-dimensional cross-linked systems that are made of a water-soluble polymer with characteristics enabling them to be suitable for use in biomedical technologies [8,9,10]. They have shown reversible responses to various stimuli such as pH, temperature, and biological molecules [11,12,13,14,15,16,17,18]. Devices that rely on the optical response are both cost-effective and durable [19,20]. pH-responsive hydrogels gained attention because of their ease of manufacturing, small size, and their biocompatibility [21,22,23]. pH-responsive hydrogels contain acidic or basic groups that respond to the pH changes [9]. The selection of the polymer is critical because the delamination of the backbone polymer affects the quality and stability of the sensor. Generally, the ionisable portion of the hydrogel determines the sensitivity and the working range of the pH sensor [20]. The sensor’s mechanical properties and sensitivity can be controlled by modifying the ratio between the polymer and the ionisable portion or the cross-linking. Fibre-optic probes that are based on hydrogels are ideal for remote and continuous monitoring of the pH levels. Their working principle relies on the change of the refractive index of the hydrogel in the presence of different pH levels, which occurs due to the volumetric shift of the hydrogel network [24,25]. 

In the past decades, pH-responsive hydrogels have been widely investigated for implementation in many applications. Every hydrogel sensor has its unique characteristics and working range due to the various formulations of hydrogels, which provide an enormous range of applications. For instant, Tamayol et al. reported a pH hydrogel sensor for epidermal wound monitoring [26]. The pH sensor changed its colour with the wound site pH, reflecting its health condition. Moreover, hydrogel-based pH sensors have been developed for drug delivery. pH sensors have been synthesised from different groups of polymers such as 2-hydroxymethacrylate, N,N’-methylenebisacrylamide, and N-isopropylacrylamide [27,28,29]. Recently, hydrogel-based pH sensors have been used for controlling insulin delivery [30,31,32].

In this work, we present a novel optical hydrogel sensor to monitor pH levels. The sensor is rapid to fabricate, cost-effective, and reusable. A Fresnel lens pattern was replicated on a pH-sensitive hydrogel in order to monitor its volumetric changes in response to the pH changes. The pH-responsive hydrogel underwent volumetric shifts with pH, i.e., the sensor expanded when pH increased and shrunk with deceasing pH levels. Consequently, the dimensions and the refractive index of the imprinted Fresnel lens changed, inducing shifts in the optical signal received from the sensor, and this is the working principle of the sensor. The sensor was tested for detecting pH in the range of 4.5–7.0. The sensor’s performance was examined under the optical microscope and by using a photodetector. Testing of the sensor was carried out in the temperature range of 20–40 °C to consider the temperature influence. 

## 2. Methodology

### 2.1. Materials

A Fresnel lens made of acrylic polymer was purchased from Thorlabs. The lens has a focal length of 32 mm, diameter of 25 mm, and 1.5 mm thickness. Hydroxyethyl methacrylate (HEMA), ethylene glycol diamethacrylate (EGDMA), acrylic acid (AA), 2-dimethoxy-2-phenylacetophenone (DMPA), sodium phosphate monobasic, sodium phosphate dibasic, isopropanol, and 3-(trimethoxysilyl) propyl methacrylate (98%) were purchased from Sigma-Aldrich and were used without further purification. 

### 2.2. Fabrication of the pH Sensor

**Figure 1:**
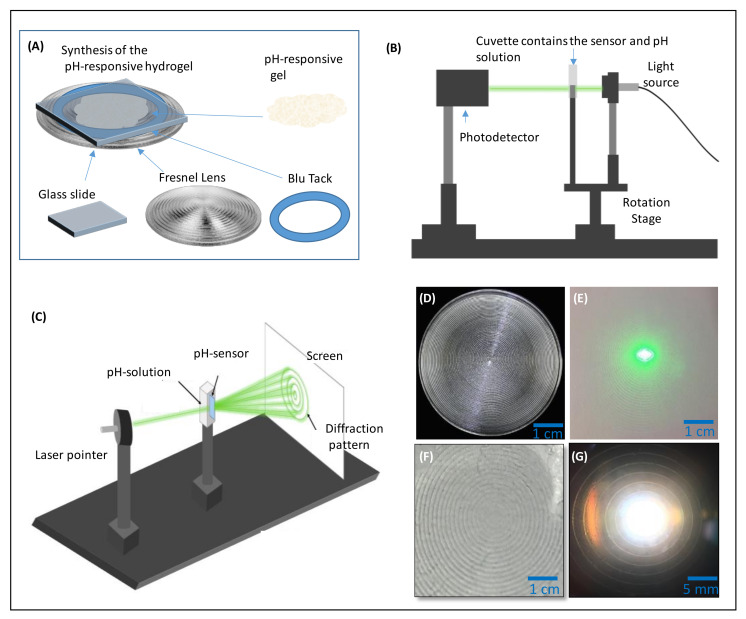
(**A**) A schematic illustration of the fabrication process for replication of the Fresnel lens on the pH-sensitive hydrogel. (**B**,**C**) schematics for the setups used to interrogate the sensor. (**D**) a photo of the master Fresnel lens that was replicated on the pH-responsive hydrogel. (**E**) a photo shows the diffraction pattern for the laser beam passed through the Fresnel lens. (**F**) a microscopic image of the surface of the pH-responsive hydrogel imprinted with the Fresnel lens, and (**G**) a photo of the pH sensor illuminated by a white light beam.

A mixture of HEMA (91.5 mol%), EGDMA (2.5 mol%), and AA (6%) was prepared. The photoinitiator (DMPA) was mixed with isopropanol in a separate plastic vial. Both mixtures were added together to form the pH-responsive gel. Approximately 40 µL of this gel was dropped on the Fresnel lens using a pipette, and a glass piece was gently placed on top (Figure 1A). The gel was polymerised using a UV light lamp of wavelength 365 nm for 5 min. The sample was kept in DI water for up to two hours to facilitate separating the sample from the master mould. When preparing the substrate-attached sensor, a salinized glass piece was used instead of the untreated one that was used in preparing the free-standing sensor. Blu Tack was used to close some grooves of the Fresnel lens to prevent any leakage of the solution. All samples were hydrated overnight in phosphate-buffered saline (PBS) solution at pH 7.4 before any further experiment. A photo of the used master Fresnel lens is shown in Figure 1D. The laser passing through the developed pH-responsive sensor showed a diffraction pattern confirming replication of the master Fresnel lens on the pH-responsive hydrogel (Figure 1E). Moreover, the images taken under the optical microscope showed the successful replication of the Fresnel structure (Figure 1F). Upon illuminating the developed sensor with white light, the Fresnel structure was quite clear (Figure 1G).

### 2.3. Measurement Techniques

A computer-controlled rotation stage, laser pointer of wavelength 532 nm, and an optical power meter (Newport, 1918-R) were used for angle-resolved optical power intensity measurements (Figure 1B). The pH sensor was fitted in a transparent plastic cuvette filled with PBS solution, ensuring that the whole sample was submerged in the solution and aligned with the laser beam and the photodetector. Then, the test started with replacing the PBS solution with the solution of pH 4.5. The pH sensor was illuminated with the laser beam, which was incident normally to the sensor’s surface plane. The laser transmitted beam was collected by rotating the sensor’s holder at 1° increments from 0° to 180°, and the photodetector recorded the light intensity at each angle. The photodetector used in this study has an aperture size of 1 cm diameter, and the laser beam used to illuminate the sensor has a diameter of 0.5 cm. The laser beam, the centre point of the sensor, and the photodetector, had to be all well aligned, so the detector could record the power for the centre of the laser diffracted beam. In case of the used photodetector having a smaller active area, the laser beam’s diameter can be reduced by using the laser aperture. Fixing the photodetector at the focal point of the sensor is another solution if a tiny photodetector is used, where the beam diameter at the focal point would be in the millimetre scale. It was most important to perform the alignment for the sensor’s central point with the laser beam and the photodetector, as it was found that it does not matter how much area the photodetector covers from the laser beam or the total illuminated area as long as it records the zero-order spot or the central point of the beam. The same steps were repeated for all tested pH solutions. In addition, an optical microscope (Zeiss) was used to measure the changes in dimensions of the Fresnel lens imprinted on the pH sensitive hydrogel while the sensor was exposed to volumetric shifts in different pH solutions. The pH sensor was placed in a transparent petri dish containing 1 mL of the tested pH solution, and the sensor was permitted to shrink and expand naturally in the solution. 

## 3. Results and Discussion

**Figure 2:**
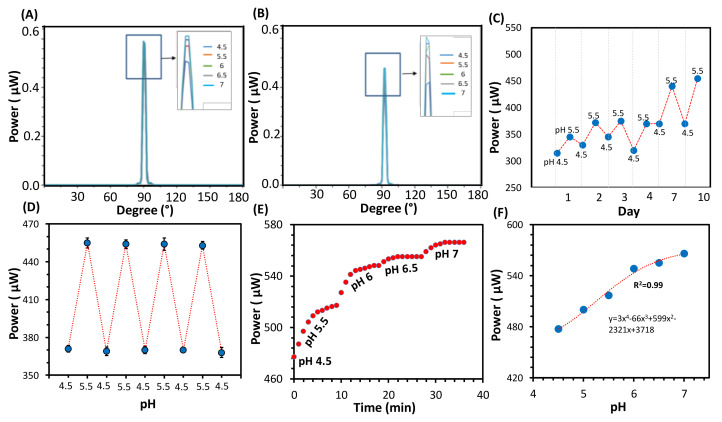
(**A**) The spatial profile of the measured power intensity for the pH sensor immersed in different pH solutions. (**B**) the spatial profile for the laser beam passed through the pH sensor that was stored for 7 days before testing. (**C**) the response on daily basis for the pH sensor interrogated in pH 4.5 and 5.5, for the first 10 days while the sensor was stored in PBS buffer in the intervals between the tests. (**D**) the optical power versus the pH for the sensor while it was immersed in pH 4.5 and 5.5 for four cycles; the standard deviation error bars calculated for *n* = 3. (**E**) pH continual detection carried out over time in different pH solutions, and (**F**) the recorded power versus the pH levels while the sensor was tested in the continuous pH detection mode.

The response of the fabricated pH sensors was investigated by detecting the spatial profile for the laser beam passing through the sensor while the sensor was immersed in different pH solutions. A schematic illustration of the used setup is shown in Figure 1B. Introducing different pH solutions in the pH range of 4.5–7.0 to the sensor led to changes in the recoded optical signals, and the maximum detected optical power increased with pH levels (Figure 2A). The sensor expanded upon increasing the pH of the tested solution due to the induced Donnan’s potential in the hydrogel matrix, resulting from ionization of the carboxyl group. The sensor showed the highest sensitivity in the pH range of 4.5–5.5 as the power increased to a value of 45 µW, which represents 7% of the reference power, and the sensitivity decreased to 6.5% and to 2.6% in the pH ranges of 5.5–6.0 and 6.5–7.0, respectively. The sensitivity of the sensor was calculated using the following formula: *S* = (ΔP/Δ_pH_) × 100, ΔP = P_pH_ − P_r_/P_r_, where *S* refers to the sensitivity, P_pH_ represents the optical power recorded for the sensor immersed in a solution of a certain pH value, P_r_ refers to the optical power recorded for the sensor when it was immersed in the reference pH solution, which in this study was considered to be the solution of pH 4.5, Δ_pH_ denotes the change in pH values. 

In order to study the effect of the shelf life on the sensor and the equilibrium period needed for the sensor to function conveniently and consistently, the pH sensor was stored in PBS solution for 7 days and the test was repeated. Again, the recorded optical signals increased with pH; however, the sensitivity of the sensor was doubled as the sensor showed an increase of 13% in the recorded power in the same pH range (4.5–5.5) (Figure 2B). To determine the equilibrium time needed for the sensor to detect pH consistently and accurately, the sensor was examined daily for 10 days while it was stored in the PBS buffer solution in the intervals between the tests. The sensor’s interrogation was carried out by illumining the sensor by a laser beam of wavelength 532 nm, and the transmitted signals were collected using a photodetector placed at a 20 cm distance from the sensor (Figure 1C). The sensor was examined in the solutions of pH 4.5 and 5.5, followed by washing and storing in the PBS buffer. It was found that the sensitivity increased with longer storage time (Figure 2C). The maximum sensitivity was achieved after storing the sensor for 10 days in the buffer. Therefore, the sensor was re-interrogated for four cycles in solutions of pH 4.5 and 5.5 (Figure 2D). The measured optical power increased from 477 µW to 454 µW upon increasing pH from 4.5 to 5.5 through the four cycles. These results indicate that the developed sensor needs to be stored in the buffer solution for 10 days before usage to provide a consistent and accurate response. 

For continual pH detection, the sensor was immersed in the pH solution and the transmitted power was recoded over time till the readings stabilized, followed by replacing the pH solution with another one of a higher pH value (Figure 2E). The response to the pH changes was immediate, and the sensor reached saturation within 9 min (Figure 2E). However, the response and saturation times of the stimuli-responsive hydrogels, in general depends on the thickness of the hydrogel sensor. Therefore, developing thinner pH sensors may achieve better performance in terms of the response and saturation times. The continuous pH detection results taken by recording the optical power of the beam supported the results obtained by recording the spatial profile of the laser beam (Figure 2B and F). The response trend of the sensor for the pH changes was nonlinear as the sensitivity was high in the pH range of 4.5–5.5, reaching 7.3% pH^−1^, and decreased slightly to 6.5% pH^−1^ at the pH range of 5.5–6.5; a slight response was detected in the pH range of 6.5–7.0 as the output signal shifted by 2.5% (Figure 2F). The output signal changed by 16.3% (90 µW) in the sensor’s working range, 4.5–7.0. The output signal changes with pH were quite easily detected using handheld digital powermeter, which cost USD 200 to 300. On the other hand, many pH hydrogel sensors have been developed based on light diffraction. Many of these sensors showed a slight shift in the diffracted wavelength with the pH changes in the acidic region (Table 1). For instance, a hydrogel sensor based on poly (ethylene glycol) diacrylate showed a sensitivity of 0.34 nm/pH, which indicates that the shift in the diffracted wavelength for the acidic region 4.5–7.0 would be 0.85 nm, less than 1 nm [33]. It is hard to detect this minute wavelength shift; in addition, the sensor’s signals need to be processed to accurately detect the wavelength shift. Moreover, a high-resolution spectrometer is needed to read the sensor, which may cost USD 2000 to 4000. The latter adds extra cost to the sensor. Performances of some pH hydrogel sensors that are based on light diffraction are given in Table 1. Their wavelength shifts with the pH changes in the acidic region of pH 4.5–7.0 (the working range of the developed sensor) are at maximum 1 nm. 

**Figure 3:**
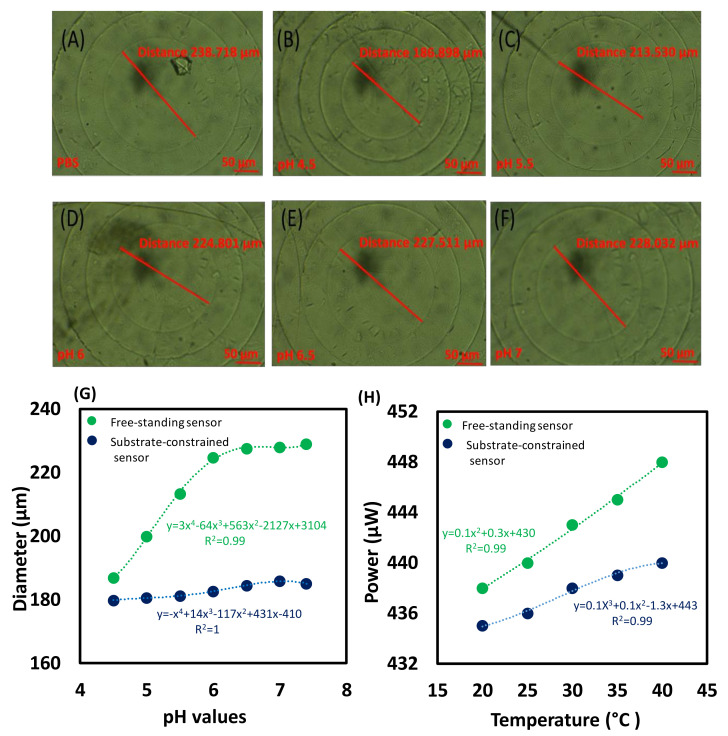
(**A**–**F**) Images taken by the optical microscope for the free-standing pH sensor immersed in different pH solutions, (**G**) diameter of the first ring of the imprinted Fresnel lens when the sensor was immersed in different pH solutions, and (**H**) the impact of the temperature on the pH sensors.

The optical microscope was used to measure and analyse the Fresnel lens dimensions replicated on the pH-responsive hydrogel, which may confirm and prove the working principle of the sensor. The test was carried out on a free-standing pH sensor and on a substrate-attached pH sensor. The cuvette contained the pH sensor was placed horizontally under the microscope to measure the change in dimensions of the Fresnel lens replicated on the sensor’s surface when the sensor was soaked in different pH solutions. The microscope images of the free-standing pH sensor immersed in different pH solutions are presented in Figure 3A–F. The images show that changing pH induced a significant volumetric shift for the hydrogel sensor. For the substrate-constrained sensor, the diameter of Fresnel lens rings slightly increased ≈6 µm, from its original value of 179 nm to 185 nm, upon increasing the pH from 4.5 to 7.0, which represents 3% of the ring diameter (Figure 3G). For the free-standing sensor, the diameter of the Fresnel lens’s ring was measured under the microscope, and the measured diameter was 238 µm at pH 7 as compared to 187 µm at pH 4.7. The initial diameter of Fresnel lens ring at pH of 4.5 was ≈187 µm, and at pH of 5.5, the diameter dramatically increased by 11%, reaching up to ≈213 µm (Figure 3G). At pH 7.0 the measured diameter value increased by ≈18%. Noticeably, the diameter of Fresnel lens’s ring rapidly dropped when the pH changed from high pH to low pH level of 4.5. Overall, the results showed that the substrate-constrained sensor had a much smaller diameter change comparing to the free-standing one. Moreover, the free-standing sensor demonstrated higher sensitivity with a significant rise in dimensions of Fresnel lens with pH.

Effect of temperature on the sensor was studied (Figure 3H). The sensor was soaked in PBS solution, and the temperature was increased from 20 to 40 °C and the transmitted optical powers were recorded. Both the substrate-constrained sensor and the free-standing sensor showed that the temperature changes in that range had no significant impact on their sensitivity. The power increased by 0.5% and 1% for the substrate-attached and the free-standing sensors, respectively. 

As the developed sensor functions in the acidic region, it may be suitable for applications in food quality, such as milk quality, as the milk pH lies in the range of 4.6–6.7 [37]. Moreover, it can be candidate for gastric pH detection when it is loaded on an optical fibre’s tip as the physiological pH of the gastric is in the range of 1–7 [38]. However, the working window of the sensor can be tuned to function in the alkaline pH region by replacing the ionisable co-monomer (AA) with 2-(dimethylamino)-ethylmethacrylate (pK_a_ = 8.4) [39]. In this case, the sensor may have a potential application in blood pH detection as the physiological pH of blood is 7.4 [16]. It is noteworthy that the developed sensor in its current designed does not fit for in situ pH monitoring as the design is not compact or portable, and requires alignments. However, attaching the sensor to the tip of a silica fibre and connecting the fibre with a three-terminal bundle-fibre that guides the laser light to the sensor and delivers the reflected power back to the photodetector would assist to avoid the needed alignments and enhance the sensor practicability. 

As compared to the SPR-based pH sensors, the developed sensor does not require costly or bulky equipment such as spectrophotometers and computers. Unlike the fluorescent pH sensors, the sensor is not prone for photobleaching and is less costly. In contrast to the holographic pH sensors, the proposed sensor is fabricated in a single-stage process without need for advanced techniques (nanosecond laser) or multistage chemical processes. Additionally, the imprinted Fresnel lens on the pH-responsive hydrogel increases the sensor’s active area and enhances the diffusion rate of the analyte into the hydrogel matrix.

## 4. Conclusions

We have demonstrated a novel pH optical sensor based on a combination of Fresnel lens and a pH-responsive hydrogel. The optical sensor has a high sensitivity in the working range of 4.5–7, and recorded a saturation time of 9 min. The sensor showed the highest sensitivity in the pH range of 4.5–5.5, and the sensitivity increased upon storing for 10 days in a PBS buffer solution of pH 7.4. The sensor showed a consistent response after storing in the buffer for 10 days. The free-standing sensor showed a three-fold sensitivity compared to that for the substrate-attached one. The temperature changes in the range of 20–40 °C were found to have a negligible effect on the sensor. Moreover, we showed that the fabrication process of such sensors is fast and cost-effective compared to their conventional counterpart, and it could be ideal for mass production. Optimizing the sensor sensitivity to function perfectly at the physiological pH range may make the developed sensors a candidate for monitoring the pH of human blood. 

## Figures and Tables

**Table 1:** Performance of the developed sensor compared to existing sensors.

pH-Responsive Hydrogel	pH Range	Sensitivity	Ref.
Poly(hydroxyethyl methacrylate)/methacrylic acid (pHEMA/MAA)	4–7	0.07 nm/pH	[34]
Poly(acrylic acid)/poly (allylamine hydrochloride)	3–7	0.45 a.u/pH	[35]
Poly(ethylene glycol diacrylate)	2–6.5	–0.41 nm/pH	[33]
Poly(hydroxyethyl methacrylate)/methacrylic acid	1–7	0.30 nm/pH	[36]
pHEMA/poly(acrylic acid)	4.5–7	6.5%/pH or 36 µW/pH	This study

## Data Availability

Not applicable.
